# Changes in hydrodynamics and water quality of a subtropical meromictic urban lake

**DOI:** 10.1007/s10661-025-14347-1

**Published:** 2025-07-15

**Authors:** Hengyu Wei, Hong Zhang, David P. Hamilton, Fuxin Zhang, Steven McVeigh

**Affiliations:** 1 School of Engineering and Built Environment, Griffith University, Gold Coast Campus, Gold Coast, QLD 4222 Australia; 2 Australian Rivers Institute, Griffith University, Nathan Campus, Brisbane, QLD 4111 Australia; 3 School of Marine Science and Engineering, South China University of Technology, Guangzhou, 511442 China; 4 City of Gold Coast, Gold Coast, QLD 4211 Australia

**Keywords:** Coastal lake, Saltwater intrusion, Meromixis, Artificial urban lake, Climate change, Stratification

## Abstract

Coastal canal and waterway development has expanded rapidly over the past half-century, contributing to environmental issues such as declining water quality and saltwater intrusion. This study analysed 35 years of field sampling data from a subtropical meromictic lake to investigate water quality changes caused by intermittent saltwater intrusion. Saltwater intrusion began after a canal expansion converted a stormwater pipe into a bidirectional conduit, allowing saltwater to flow into the lake during high tides. Lake surface water temperature increased at a rate of 0.019 °C p.a. and pH has shifted from acidic to circumneutral over the study period, likely due to the disturbance of a pyrite layer during the initial construction phase and progressive increases in trophic state. Salinity accumulation in the bottom waters has caused the lake to transition from monomictic to meromictic, with stratification increasing through the 1990s, as evidenced by increases in Schmidt stability index. This shift to meromixis has resulted in permanent bottom-water deoxygenation and has led to eutrophication, evidenced by elevated nutrient levels and the development of a deep chlorophyll layer at the chemocline of the lake within recent years. This study provides valuable insights for managing other coastal lakes and wetlands experiencing similar challenges, offering guidance on mitigating the adverse impacts of saltwater intrusion that may increase with sea level rise due to climate change.

## Introduction

Artificial coastal lakes are human-made bodies of standing water located along the coastline. These lakes are typically created from former coastal wetlands for various purposes, including environmental conservation, recreational activities, flood control, and economic development (Johnson & Williams, 1989; Waltham & Connolly, 2011, 2013). With urban development and increasing human populations in coastal habitats worldwide, many coastal artificial lakes have been constructed since the mid- twentieth century (Kennish, 2002). Waltham and Connolly (2011) estimated that the global artificial residential waterways had reached a total of 4000 linear km, covering an area of 270 km^2^ by 2011. However, these artificial lakes differ significantly from the shallow wetland habitats they replace (Morton, 1992). They are usually dredged, and sometimes the underlying soil is disturbed, leading to the formation of sulfuric acid from acid-sulphate soils (Waltham & Connolly, 2011). In addition, with predictions of ongoing increases in population in coastal regions, the ecosystems of these coastal artificial lakes are facing profound challenges from climate change, urbanisation, and eutrophication (Reimann et al., 2023; Steven et al., 2023).

Freshwater lakes in lowland coastal regions may be subject to periodic saltwater intrusion during high tides, storm surges, or morphological changes. For example, Lake Pontchartrain has been influenced by saltwater intrusion due to the construction of a navigation canal to the Mississippi River estuary, leading to occasional localised water column stratification, as observed by Stern and Stern (1969). The canal was later closed in 2009, and subsequent research found that the closure had stopped saltwater intrusion and associated low dissolved oxygen (DO) levels in bottom waters (Poirrier, 2013). Thienpont et al. (2012) investigated the paleo record of five lakes of the Mackenzie Delta and discovered varying levels of saltwater intrusion in these lakes due to a marine stormwater surge. Increased salinity in freshwater-dependent coastal lakes may alter biogeochemical cycles and harm freshwater biota (Bhattachan et al., 2018; Junot et al., 1983). Sea level rise driven by climate change is expected to initiate or accelerate saltwater intrusion and affect water quality (Bhattachan et al., 2018; Kivilä et al., 2022; Thienpont et al., 2012).

Saltwater intrusion can affect vertical density gradient in lakes, which drives the development of chemical transformations that affect flora and fauna (Boehrer & Schultze, 2008; Boehrer et al., 2017; Krasnova, 2021). Lake Fidler, in Tasmania, Australia, is a small meromictic lake that was subject to intermittent inundation of brackish water due to tidal incursions from the adjacent Gordon River. Hodgson et al. (1997) found that thermal stability in the lake was reduced because the construction of the Gordon River dam downstream of the lake in 1977 prevented incursion of brackish water into the lake. The decline in meromictic stability led to significant biological changes in the lake and the elimination of many microorganisms associated with the meromictic state (Hodgson & Tyler, 1996).

Freshwater coastal lakes vulnerable to saltwater intrusion may respond with changes to vertical density gradients, causing prolonged stratification periods or even an entirely altered mixing regime. To date, only a few cases of transitions in stratification regime have been reported in coastal lakes. These transitions may have occurred abruptly thousands of years ago (Thienpont et al., 2012) or recently in response to human actions. To assess the environmental effects of such transitions, it is essential to understand the mechanisms behind them, including how meromixis develops and is sustained (Boehrer & Schultze, 2008). We investigated an Australian subtropical meromictic coastal lake with 35 years of historical monitoring data, which underwent a mixing regime transition from holomixis to meromixis in recent years. This research aims to (i) identify the effects of saltwater intrusion on the lake mixing regime, (ii) assess the influence of the changed mixing regime on the water quality, (iii) explore the potential consequences of climate change on both the mixing regime and water quality of the lake.

## Methodology

### Study area

**Fig. 1:**
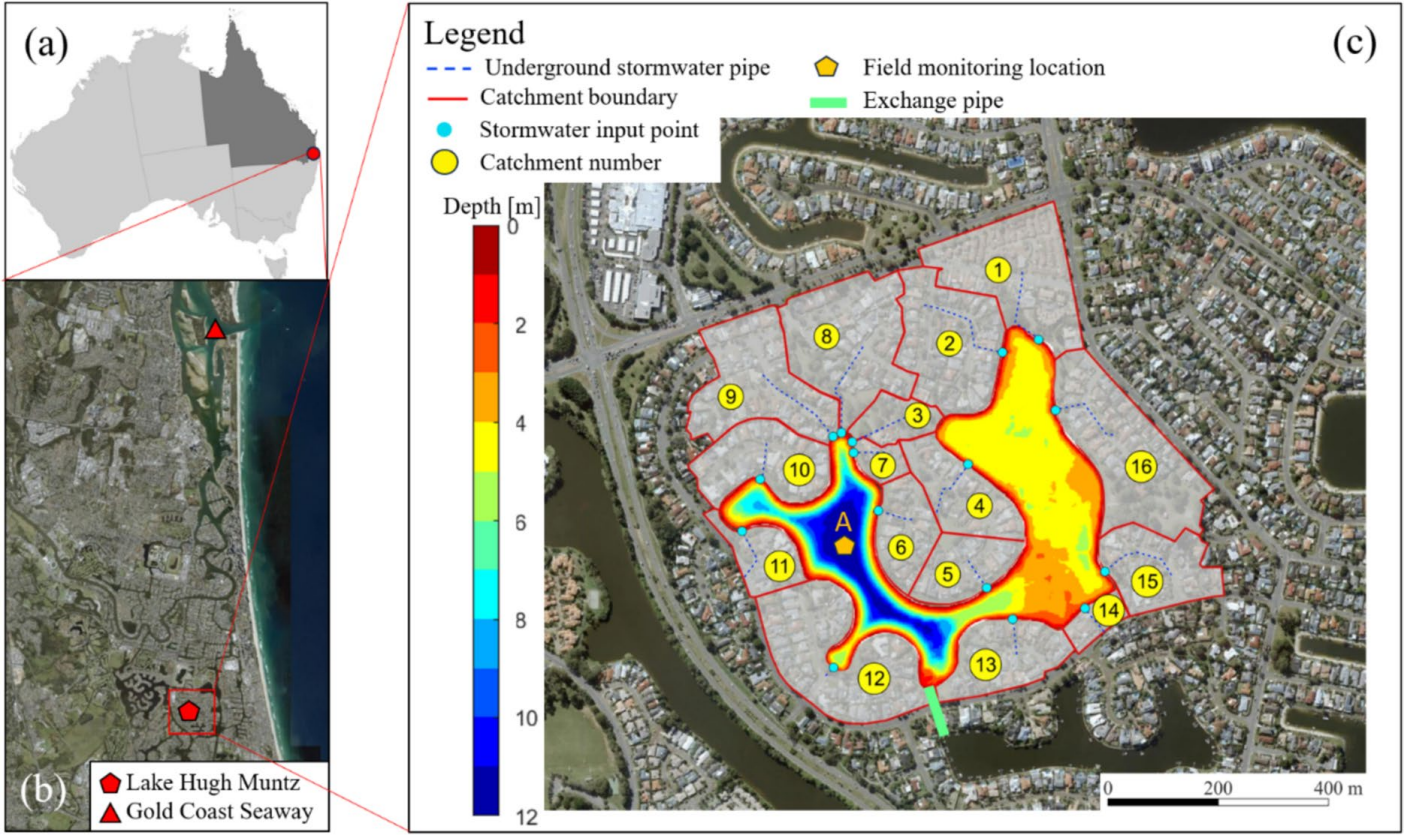
**a** Australia and Queensland **b** Satellite imagery of Gold Coast (Source: Queensland Globe Imagery), showing the geographic location of LHM, and Gold Coast Seaway; **c** Satellite imagery, bathymetry of LHM, City of Gold Coast (CoGC) field monitoring and sampling location A, 16 stormwater catchments (1–16), and the exchange pipe location (at the southmost point of the lake) (catchment map revised from Brushett et al. (2008)

Lake Hugh Muntz (LHM) is an artificial lake located at Gold Coast, Australia, with a surface area of 15 ha, a maximum depth of 12 m, and a volume of approximately 800 ML (Fig. 1). The region’s climate is characterised as subtropical, with an annual mean rainfall of about 1300 mm. Typically, the minimum monthly rainfall occurs in September (43.4 mm) and the maximum in February (189.0 mm). The hottest month is January, with an average daily temperature of 25.3 °C, while the coldest month is July, with an average daily temperature of 16.7 °C.

The City of Gold Coast has an extensive network of waterways comprising rivers, creeks, urban lakes, and canals. Approximately 44% (14 ${\text{km}}^{2}$ out of 31 ${\text{km}}^{2}$) of the artificial lakes globally occur on the Gold Coast (Waltham & Connolly, 2011). Initially, canals were constructed to extend the natural river-estuary, but large-scale artificial lake construction replaced canal development in the late 1980 s to reduce the tidal prism in the lower Nerang estuary, thereby reducing downstream scour effects and erosion caused by tidal flow (Johnson & Williams, 1989).

**Fig. 2:**
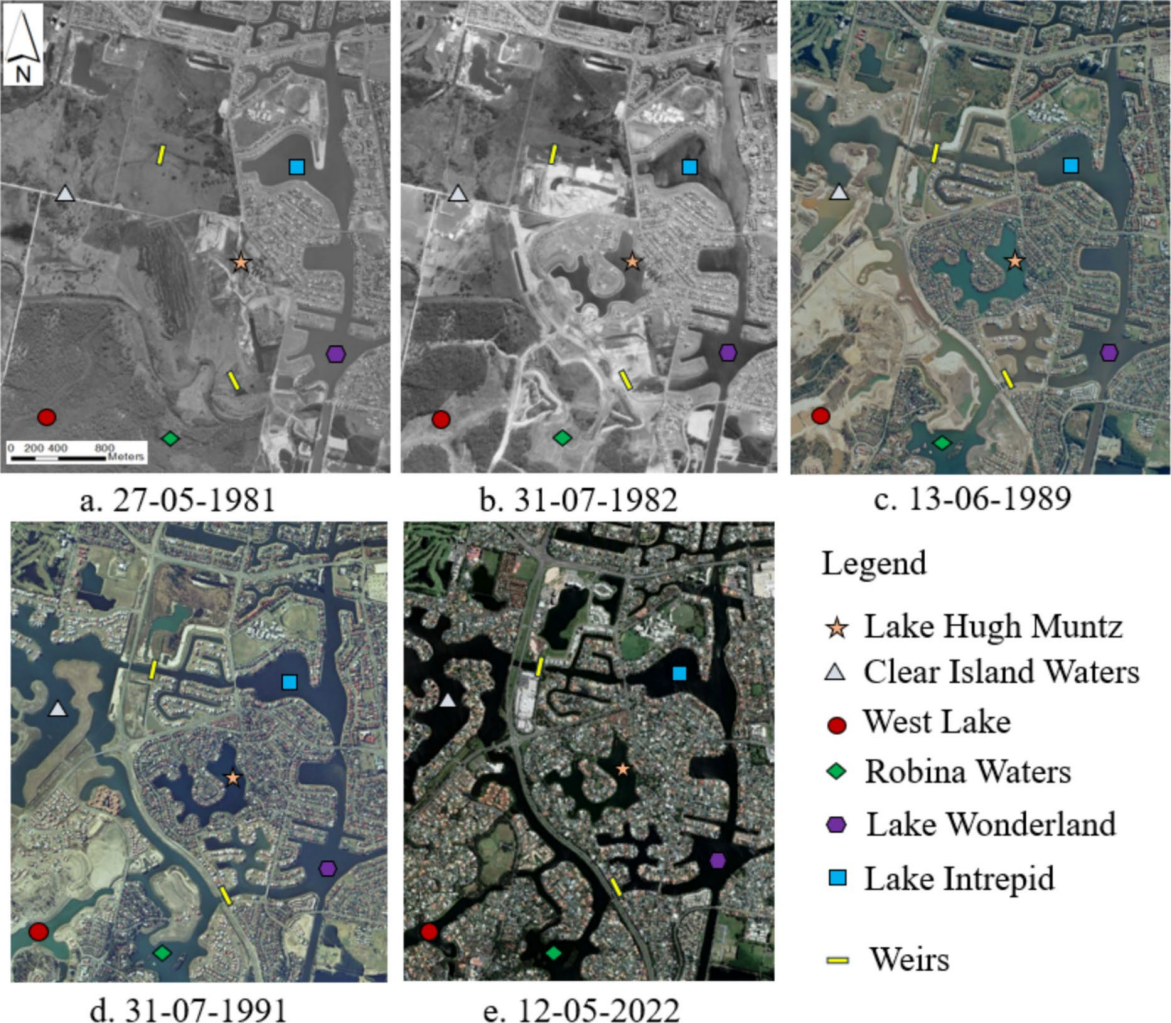
Aerial imagery of Lake Hugh Muntz and the surrounding area. **a** Construction of the lake began early in 1981, **b** the construction was finished by July 1982, **c** construction of the canal to the south of the lake was completed in 1989, with Clear Island Water Lake (northwest of LHM) still under construction, **d** construction was completed in 1991, and **e** a 2022 image of LHM (Queensland Government, 2022). Locations of surrounding lakes and weirs are marked on all five maps for comparison

LHM was constructed between 1981 and 1982 by the City of Gold Coast as a stormwater detention basin, as part of the development of an adjacent canal system which was completed in 1989 (Fig. 2). The original images have been rotated to the true north direction and cropped for uniformity. LHM and the adjacent canals and lakes were constructed in the three stages. The first stage included the initial construction of the lake (Fig. 2a, b). The second stage, completed in 1989, included the construction of a canal to the south of the lake (Fig. 2c) and a canal exchange pipe (Fig. 1c). The third stage, completed in 1991, involved the construction of additional lakes and canals to the west of LHM (Fig. 2d). LHM is indirectly connected to the Pacific Ocean via the Nerang River. Lake Wonderland and Lake Intrepid (Fig. 2) are part of the Nerang River Estuary canal system, which comprises numerous branching waterways. They lie within the tidal reach of the Nerang River estuary and are therefore subject to tidal influence. Macklin et al. (2017) reported salinity levels ranging from 16.9 to 22.6 PSU (practical salinity units) in these lakes. In contrast, Robina Waters, West Lake, and Clear Island Waters are separated from the estuary by weirs and tidal gates, and therefore are not directly influenced by tidal incursion.

The exchange pipe connecting the canal and LHM is positioned at the lake’s surface. A weir structure is in place, allowing water to overflow and enter the lake via the pipe when the water level exceeds the height of the weir. This mechanism functions in both directions: when the lake level rises above the weir, lake water overflows into the canal; conversely, during high tide, when the canal water level exceeds the weir height, high-salinity canal water flows into the lake.

Water quality of LHM has been monitored by the City of Gold Coast since 1987 and has deteriorated over the period of this study, particularly in terms of eutrophication and algal blooms (Brushett et al., 2008; Burford et al., 2018; Waltham et al., 2014).

### Monitoring data

LHM’s monitoring program can be separated into three distinct periods (Table 1). Simmonds & Bristow Pty. Ltd. conducted quarterly vertical profiling and water quality sampling from 1987 to 1997. Vertical profiling of temperature, electrical conductivity, dissolved oxygen (DO), and pH was undertaken at 1–2 m intervals through the water depth at location A (Fig. 1c), and water clarity was measured with a Secchi depth. Surface water samples (0.3 m depth) were taken at the same location for analysis of total suspended solids, turbidity, ammonium-nitrogen, and chlorophyll-a (chl-a).

**Table 1:** Lake Hugh Muntz monitoring program details for location A (see Fig. 1 for location). CoGC is City of Gold Coast

Monitoring Period	Time span	Sampling agency	Measurement frequency	Vertical profiling range (m)	Vertical interval (m)
1	1987–1997	Simmonds & Bristow Pty Ltd	Quarterly	0.3 to 11	1 or 2
2	1999–2010	CoGC	Quarterly	0.3 to 6	2
3	2010–2012	CoGC	Quarterly	0.3 to 12	1
	2012–2023	CoGC	Monthly	0.3 to 12	1

From 1999 to 2010, the City of Gold Coast (CoGC) monitored LHM quarterly, with water temperature, electrical conductivity, DO, and pH recorded at three depths using a calibrated Hydrolab (OTT HydroMet, USA) multi-probe. The three depths were not consistent during this period, and the measurements are recorded mostly for the water column above a depth of 6 m. Water samples were taken at depths of 0.3, 2, 4, and 6 m from each site for analysis of total nitrogen (TN), total phosphorus (TP), ammonium-nitrogen, oxidised nitrogen (nitrite and nitrate), total organic nitrogen, orthophosphate, chl-a, turbidity, and total suspended solids (TSS).

In 2010, CoGC updated its lake monitoring programme. Vertical profiles were taken for the entire water column from 0.3 to 12 m at 1-m intervals. From August 2012, monitoring was undertaken monthly, with samples taken for water quality analysis at 2 to 6 m vertical intervals for the same constituents as those measured from 1999 to 2010.

All sample collection and analysis procedures followed the standard methods set by APHA (American Public Health Association et al., 2023). Dissolved nutrient samples were collected in situ and filtered on site. All samples were stored on ice until they were returned to the laboratory, where they were subsequently frozen. Analyses were conducted at a laboratory accredited by the National Association of Testing Authorities (www.nata.com.au). Minor variations in analytical procedures occurred as multiple groups carried out sampling over the course of this study.

Historical monthly averaged air temperature data were collected from Gold Coast Seaway Bureau of Meteorology (BoM, http://www.bom.gov.au/) station 040764 (see Fig. 1b). However, air temperature data was not available at this station prior to 1995.

### Vertical profile analysis

Vertical profiling data of temperature, conductivity, pH, and dissolved oxygen at Location A over the past four decades were visualised as heat maps using MATLAB software. In addition, water quality parameters, including temperature, conductivity, pH, dissolved oxygen, turbidity, total phosphorus, orthophosphates, chlorophyll-a, total nitrogen, ammonium-nitrogen, total organic nitrogen, and dissolved oxidised nitrogen, were plotted as box plots at various depths to illustrate their depth distribution. On each box, the central mark indicates the median, and the bottom and top edges of the box indicate the 25th (Q25) and 75th (Q75) percentiles, respectively, and the whiskers extend to the most extreme data points not considered as outliers. Outliers were defined as values that lie more than 1.5 times the interquartile range (IQR = Q75–Q25) below the first quartile (Q25) or above the third quartile (Q75).

### Time series analysis

Time series were plotted of surface water quality parameters at location A over the study period, including total suspended solids, turbidity, chl-a, ammonium-nitrogen, total nitrogen, and total phosphorus, at location A are plotted to show the trends. Changes in surface water temperature at location A from 1987 to 2023 were examined by fitting of temperature and day-of-year to a sinusoidal model using the non-linear least squares method (Eq. 1):

1 $${T}_{surface}=A\cdot \mathit{sin}\left(\frac{2\pi \cdot }{365}{t}_{d}+B\right)+C$$

where $A$, $B,$ and $C$ are fitting parameters for the sinusoidal model; ${t}_{d}$, and ${T}_{surface}$ are day of year and surface water temperature, respectively. Residuals between the modelled (sinusoidal regression) and measured values were then calculated and plotted as time series for the monitoring period. A *p* value was calculated using the t-distribution and serves as a measure of the statistical significance of each parameter in the sinusoidal model (Wang et al., 2019), with *p* < 0.05 considered statistically significant.

### Lake stability analysis

The stability of stratification in the lake was quantified using the Schmidt stability index (SSI). SSI is a valuable tool for predicting how changes in climate or external inputs, such as stormwater inflows, might influence the lake stratification and mixing regime, and associated water quality parameters (Bertone et al., 2015; Zhang et al., 2020). SSI was calculated as follows:

2 $$SSI=\frac{g}{{A}_{0}}\underset{0}{\overset{D}{\int }}(z - {z}_{*})({\rho }_{z}-\overline{\rho }){A}_{z}dz$$

where $g$ is the gravitational acceleration, ${A}_{0}$ is the surface area of the lake, $D$ is the maximum depth of the lake, ${\rho }_{z}$ is the water density at depth $z$, and ${\rho }_{z}$ is calculated using temperature and salinity (Millero & Poisson, 1981), which was derived from water temperature and conductivity using the UNESCO equation (Fofonoff & Millard., 1983), $\overline{\rho }$ is the average water density of the lake, ${z}_{*}$ is the depth at which the value of $\overline{\rho }$ is located in the stratified density profile, and ${A}_{z}$ is the area of the lake at depth $z$.

A temperature-normalised Schmidt Stability Index (SSI-S) was calculated by assuming a uniform reference water temperature of 20 °C throughout the entire water column. This approach isolates the effect of salinity on lake stability by removing the influence of thermal stratification. This reference temperature was not used when converting conductivity to salinity, as doing so would introduce a temperature bias in the calculation of practical salinity units using the UNESCO equation. In addition to SSI-S, the effect of water temperature on stability, denoted as SSI-T, was calculated as the difference between the total SSI and SSI-S.

### Salt calculation

The total amount of salt ($M$) in the lake water at each measurement date was estimated using the following equation:

3 $$M=\underset{0}{\overset{D}{\int }}{A}_{z}{{\rho }_{z}S}_{z}dz$$

where ${S}_{z}$ is the salinity in Practical Salinity Units (PSU) derived from water temperature and conductivity at depth $z$ using the UNESCO equation (Fofonoff & Millard., 1983).

There were some limitations in the calculations of the SSI and salt mass due to data availability. The estimation process involved calculating total salt mass in kilograms for each of the 12 depth-resolved horizontal layers. Since the temperature and conductivity were not consistently available for deeper layers in the first 10 years of the study, the water properties of the deepest data-available layer were adopted. To ensure the reliability of the calculations, profiles with a maximum depth < 8 m were excluded from the analysis.

## Results

### Effects of saltwater intrusion on water quality

**Fig. 3:**
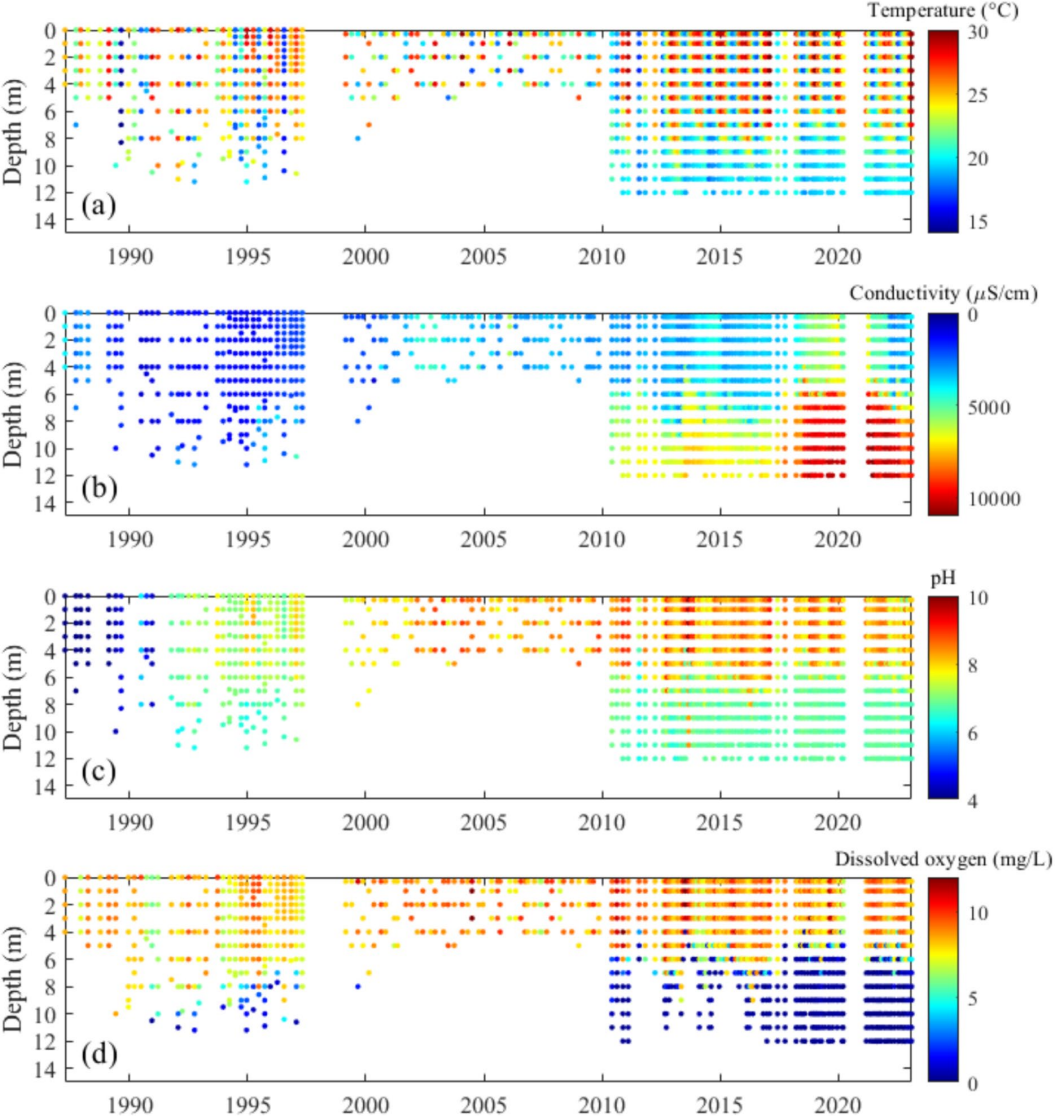
Heat maps of **a** water temperature, **b** conductivity, **c** pH, and **d** dissolved oxygen at Location A (Fig. 1b) in Lake Hugh Muntz

**Fig. 4:**
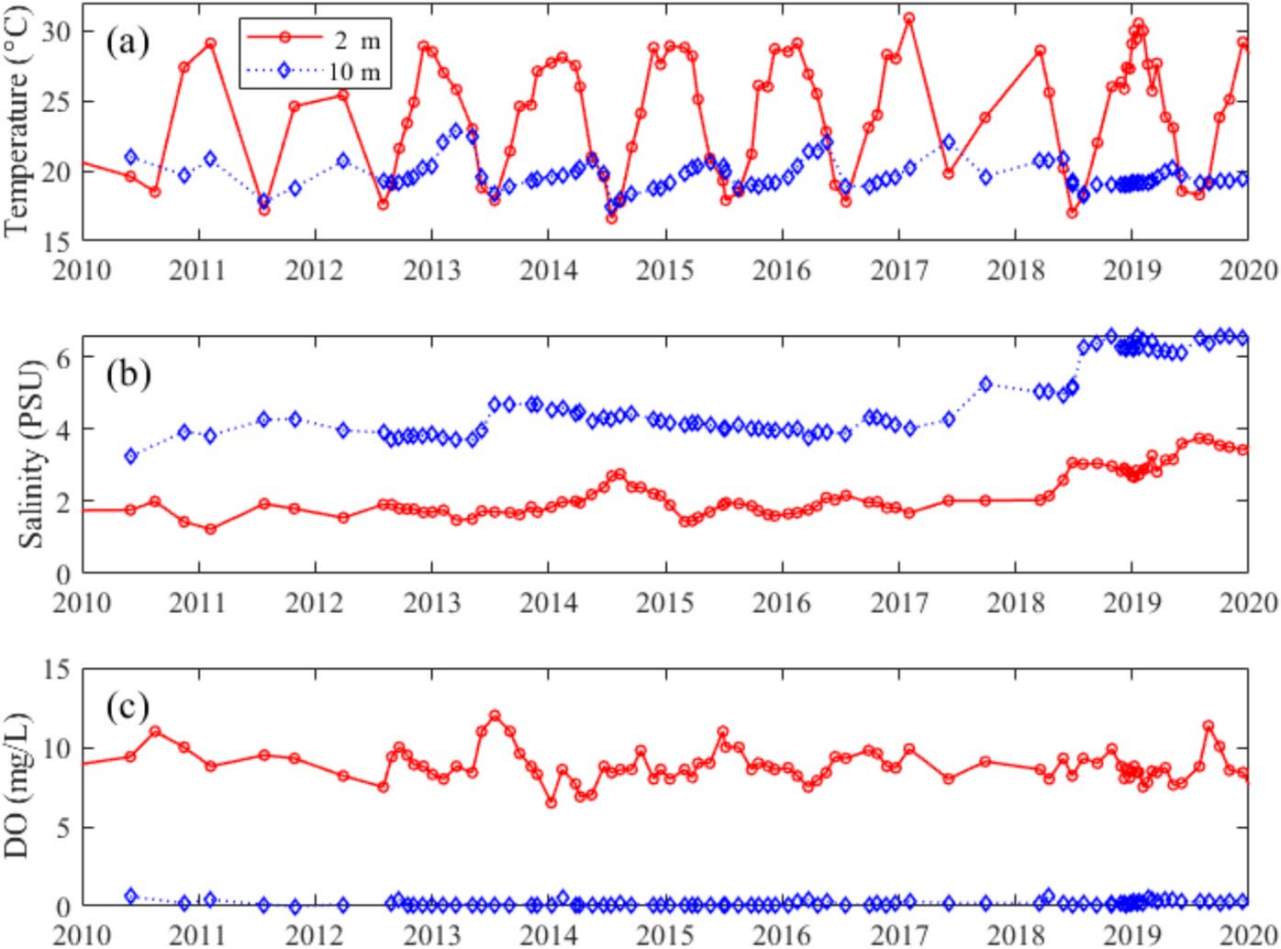
2 m and 10 m **a** water temperature, **b** salinity, and **c** dissolved oxygen at Location A (Fig. 1b) in Lake Hugh Muntz

Vertical profiles of temperature, conductivity, pH and DO at Location A (see Fig. 1) from 1987 to 2022 demonstrate increasing stratification of the water column over time (Fig. 3). The surface temperature (2 m) of LHM exhibits a clear seasonal pattern, with pronounced peaks during summer months, while the deeper layer (10 m) remains relatively stable, fluctuating slightly around 20 °C. Notably, between 2010 and 2020, there were instances when the surface temperature drops below that of the 10 m layer (Fig. 4a).

Conductivity has been steadily increasing throughout the water column of the lake, with an accelerating rate of increase after 2010 (Fig. 3b). Both the conductivity and its vertical gradient further increased after 2018 (Fig. 4b).

The pH at Location A for both top and bottom of the water column increased sharply in 1990 (Fig. 3c), the year after the completion of the canal exchange pipe. Surface pH increased from less than 4.4 to near circumneutral (6.8) in less than a year from December 1990 to October 1991 and stayed above 6.7 thereafter. A further increase in surface pH values was observed around 2002 when the surface layer started to become slightly alkaline. The vertical gradient of pH developed after the sharp increase after 1990. pH was fairly uniform through the water column from 1990 to 1997, and by the time deep profiling was reestablished in 2010, pH stratification was well established. Since 2010, the upper layer water has been slightly alkaline with some seasonal variation, while the bottom layer pH has remained below 7.0 throughout the monitoring period, with only one exception in 2014.

The bottom waters became anoxic (DO < 0.2 mg/L) as early as December 1990, less than two years after the exchange pipe was constructed. During 1991 to 1997, depths to low dissolved oxygen (< 2 mg/L) became shallower, from about 10.5 to 8 m. The stratification of dissolved oxygen was first observed in December 1990 and was clearly evident after 2010. From 2010 to 2022, the bottom water remained mostly anoxic (< 0.2 mg/L), with only a few occasions when oxygen was detected below depths of 8 m (Fig. 4c).

**Fig. 5:**
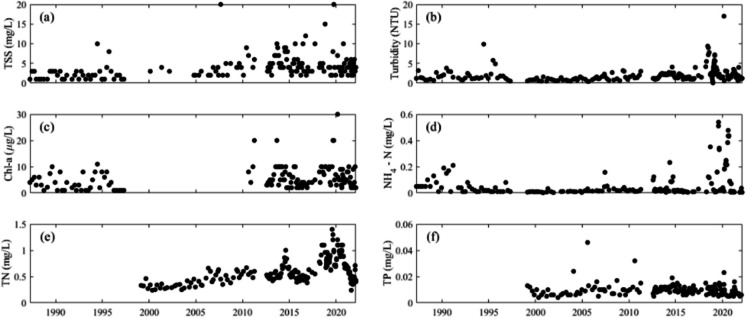
Surface (0.3 m depth) water quality parameters in Lake Hugh Muntz: **a** total suspended solids (TSS), **b** turbidity, **c** chl-a, **d** ammonium-nitrogen, **e** total nitrogen (TN), and **f** total phosphorus (TP). Note that commencement of sample analysis varies among parameters

Surface total suspended solids (TSS) increased steadily after1987 (Fig. 5) while turbidity increased after 1999. On a few occasions, surface chlorophyll-a (chl-a) was > 10 $\mu \text{g}/L$ after 2010, while no such event was observed from 1987 to 1997. Ammonium-N showed three periods of increase. The first from 1989 to 1991, the second in 2014 lasted only a few months, and the third began in 2018, increasing substantially thereafter for three years. TN increased steadily since the first observation in 1999. Peaks in TN tended to mirror those of peaks in ammonia-N. TN started to decrease after a peak in 2019. TP concentrations have been relatively stable since the first samples in 1999, with only a few isolated occurrences of concentrations > 0.02 mg/L.

### Vertical distribution of water quality

**Fig. 6:**
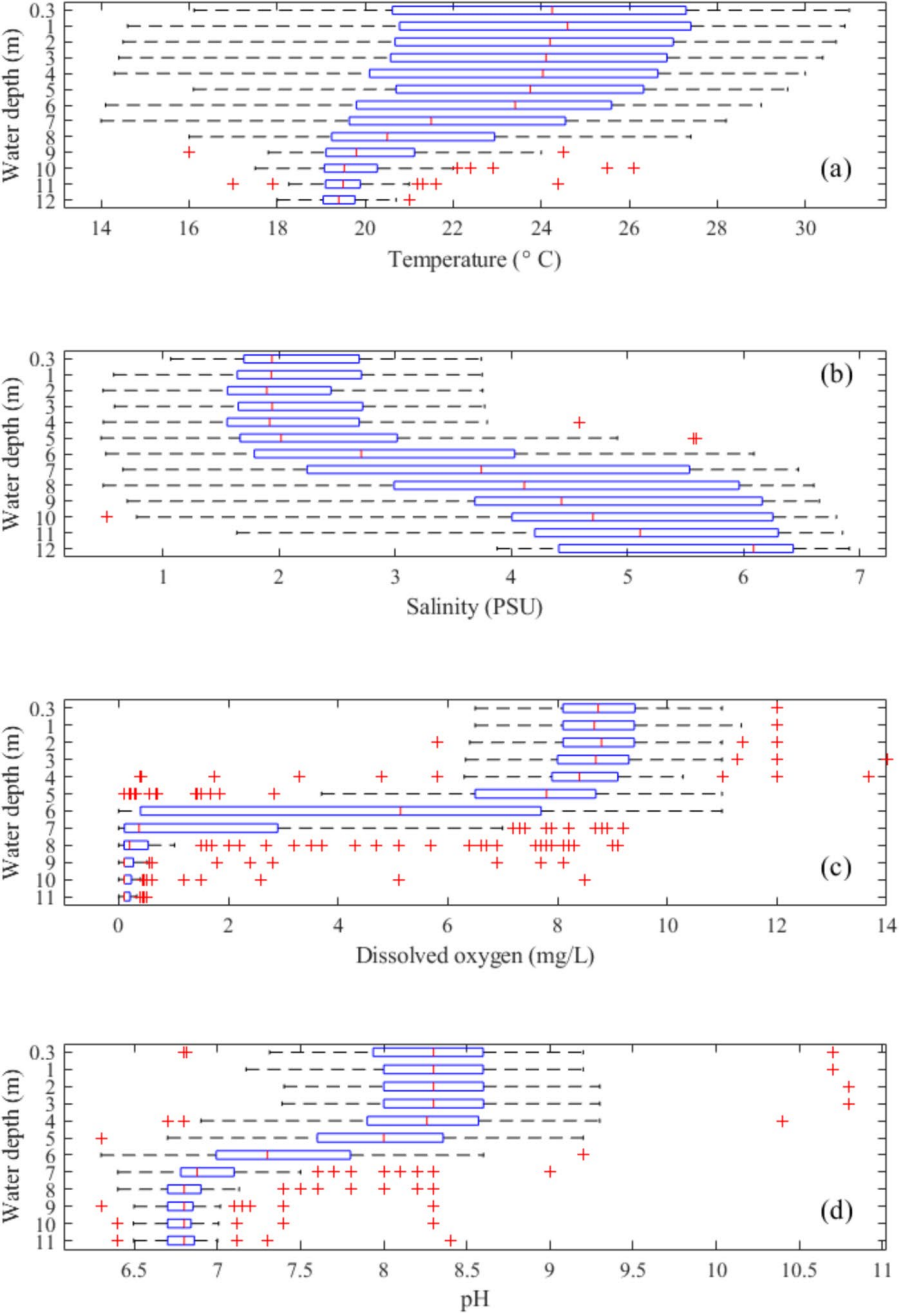
Vertical variations of **a** temperature, **b** salinity, **c** dissolved oxygen and **d** pH of LHM from 2010 to 2023. On each box, the central mark indicates the median, and the bottom and top edges of the box indicate the 25th and 75th percentiles, respectively. The whiskers extend to the most extreme data points not considered outliers, and the outliers are plotted individually using the ‘ + ’ marker symbol

Box plots are presented for water quality data at Location A from 2010 to 2023, showing vertical distributions of temperature, salinity, dissolved oxygen concentration, and pH (Fig. 6). LHM displays typical meromictic lake features of vertical salinity stratification for the past decade. The lake bottom salinity is elevated compared to surface layer values. The median salinity of the bottom layer is ~ 2.5 PSU higher than the surface layer, where median salinity is ~ 2 PSU (Table 2). Surface temperature can reach up to 31 °C during summer months and can be lower than bottom waters during June and July. A sharp transition layer, the chemocline, separates the surface oxic layer from the bottom anoxic layer. The mixolimnion is slightly alkaline, while the monimolimnion is usually acidic, with a few outliers extending into the alkaline range.

**Table 2:** Range and median (in parentheses) value of water temperature, conductivity, pH and DO of Lake Hugh Muntz at depths of 0.3, 8, and 10 from 1987 to 2023. Three different periods are separated according to Table 1

Period	Depth (m)	NOM*	Temperature (°C)	Conductivity ($\mu$S/cm)	pH	DO (mg/L)
1987–1997	0.3	36	14.7–28.7 (24.8)	800–4000 (1500)	3.3–7.9 (7)	5.8–9.9 (8.35)
	8	20	16–27.4 (23.4)	1000–3900 (1465)	4.4–7.3 (6.6)	0.5–9.1 (6.55)
	10	6	18.5–26.1 (20.6)	1060–3600 (1810)	4.3–6.8 (6.5)	1.2–8.5 (2.6)
1999–2010	0.3	40	17.3–29.8 (22.9)	2000–5950 (3190)	7.4–9.0 (8.1)	6.5–11 (8.8)
	8	1	-	-	-	-
2010–2023	0.3	119	16.7–31.0 (24.9)	2600–6920 (3800)	7.4–10.7 (8.3)	6.5–12 (8.74)
	8	118	18.0–26.7 (23.3)	2800–10,360 (7000)	6.4–8.3 (6.8)	0–8.3 (0.24)
	10	119	17.5–22.9 (19.5)	5500–10,890 (8140)	6.4–8.3 (6.8)	0–0.6 (0.2)

*NOM is the number of measurements

**Fig. 7:**
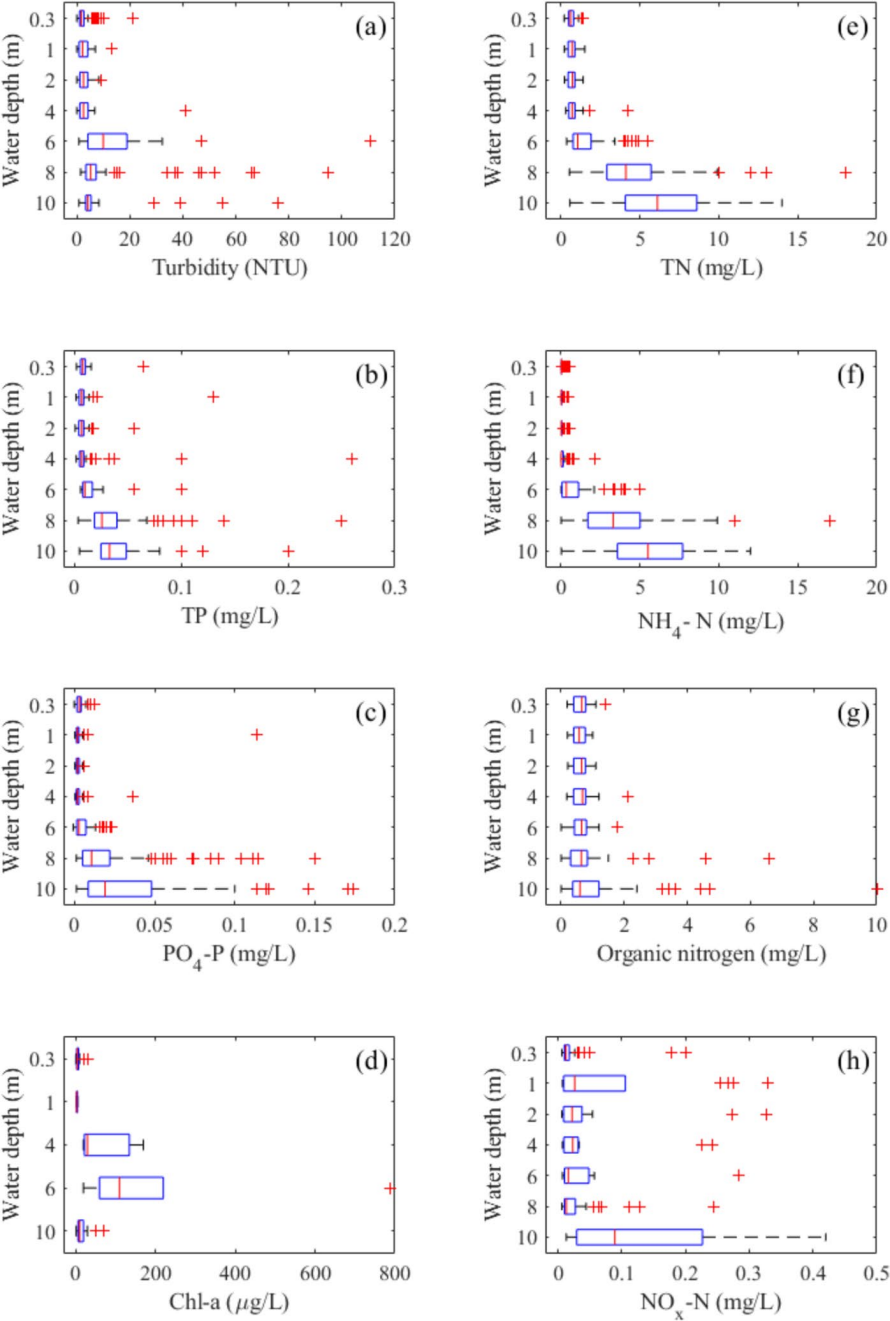
Vertical variations of **a** turbidity, **b** total phosphorus, **c** orthophosphates, **d** chl-a, **e** total nitrogen, **f** ammonium-nitrogen, **g** total organic nitrogen, and **h** dissolved oxidised nitrogen for Lake Hugh Muntz from 2010 to 2023

LHM nitrogen and phosphorus concentrations are strongly elevated in the bottom layer (Fig. 7). The median TN and TP concentrations at 10 m are 6.1 mg/L and 0.033 mg/L, respectively, while at 0.3 m, they are 0.47 mg/L and 0.008 mg/L. The nitrogen in the bottom layer is mostly ammonium, with lower concentrations of organic nitrogen and oxidised nitrogen. A high turbidity layer exists at around 6 m depth, which coincides with a deep chlorophyll layer (DCL) at around the same depth.

### Water temperature trends

**Fig. 8:**
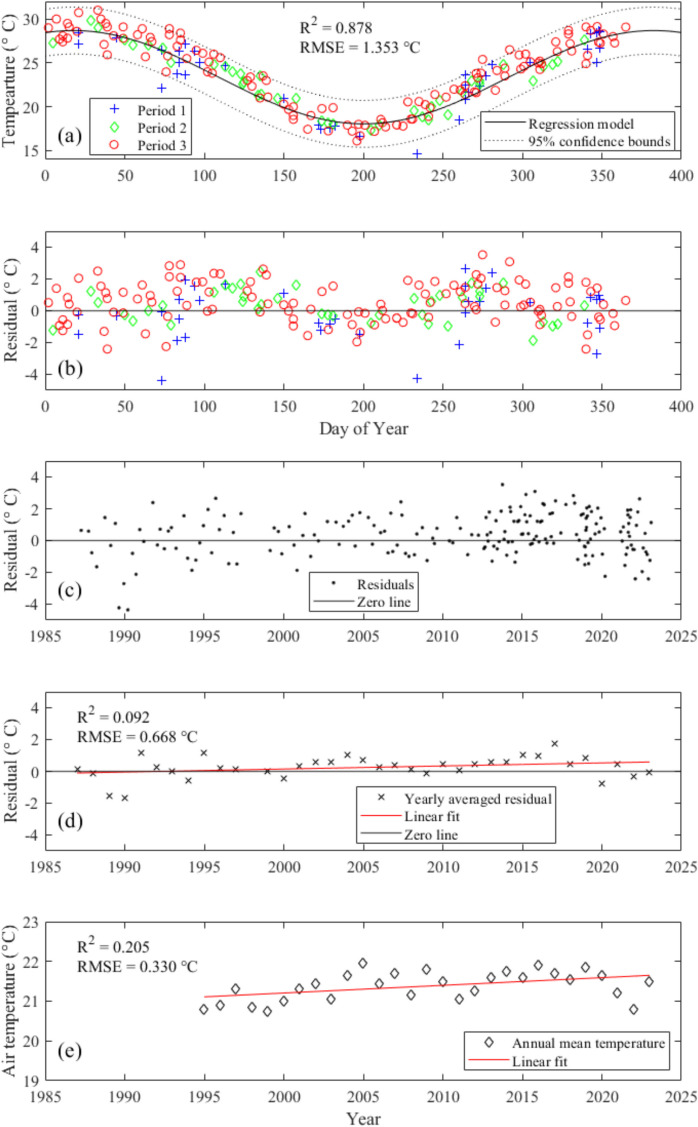
**a** Surface (0.3 m) water temperature at location A from 1987 to 2023, **b** residuals between the model prediction and observations, **c** the residuals of Fig. 8b plotted as time series and **d** yearly averaged residual, **e** yearly averaged temperature at Gold Coast Seaway. The fit in (**d**) is ${T}_{lake}=0.019Y-38.232$ and in (**e**) is ${T}_{air}=0.019Y-17.463$, where ${T}_{lake}$ and ${T}_{air}$ are the lake and air temperature, respectively, and *Y* is the year

All available historical records of surface water temperature at location A (from 1987 to 2023 exhibits clear seasonal variations, ranging from 14.5 to 30.7 °C (Fig. 8a). During summer months (Dec, Jan, Feb), the average 0.3 m water temperature is 28.2 °C, while in winter (Jun, Jul, Aug) the average is 18.4 °C. Analysis of the long historical series shows that the 0.3 m water temperature can be well fit with a sinusoidal distribution expressed as follows:

4 $${T}_{surface}=5.315\bullet \mathit{sin}\left(\frac{2\pi \bullet {t}_{d}}{365}+1.265\right)+23.370$$

$${\text{R}}^{2}=0.878,\text{ RMSE}=1.353 ^\circ \text{C}$$

The residuals between the measurements and the modelled values (Fig. 8b) were plotted as a time series (Fig. 8c) and averaged annually for the entire monitoring period (Fig. 8d), to minimise statistical bias from non-uniform monitoring intervals. A regression line to capture the variation in annual temperature residuals resulted in a regression line of 0.019 °C/yr, which was identical to the slope of the regression of annual mean air temperature for the Gold Coast Seaway (BoM station 040764) from 1995 to 2023 (Fig. 8e).

**Fig. 9:**
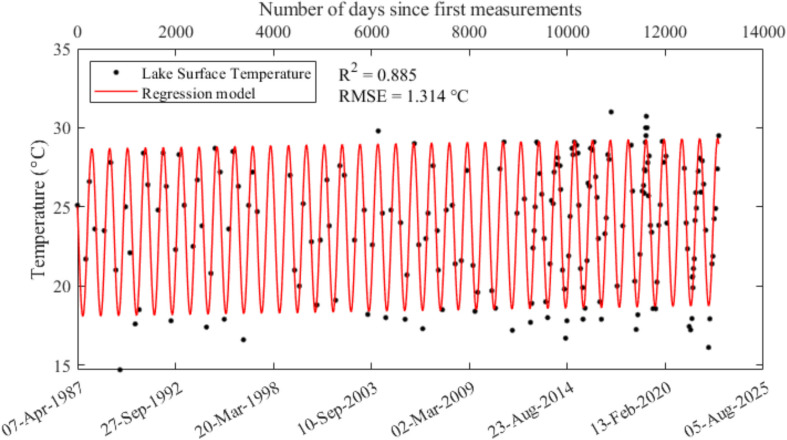
Sinusoidal regression of 0.3 m water temperature at location A from 1989 to 2023. The horizontal axis is in number of elapsed days since the first measurement

Using the 0.3 m water temperature increase of 0.019 °C/yr, the 0.3 m water temperature can be re-modelled with an additional slope factor for the entire monitoring period (Fig. 9):

5 $${T}_{surface}=5.315\bullet \mathit{sin}\left(\frac{2\pi \bullet t}{365}-2.823\right)+0.019*(\frac{t}{365}) +23.370$$

$${\text{R}}^{2}=0.885\text{ and p}<0.01$$

### Lake stability

**Fig. 10:**
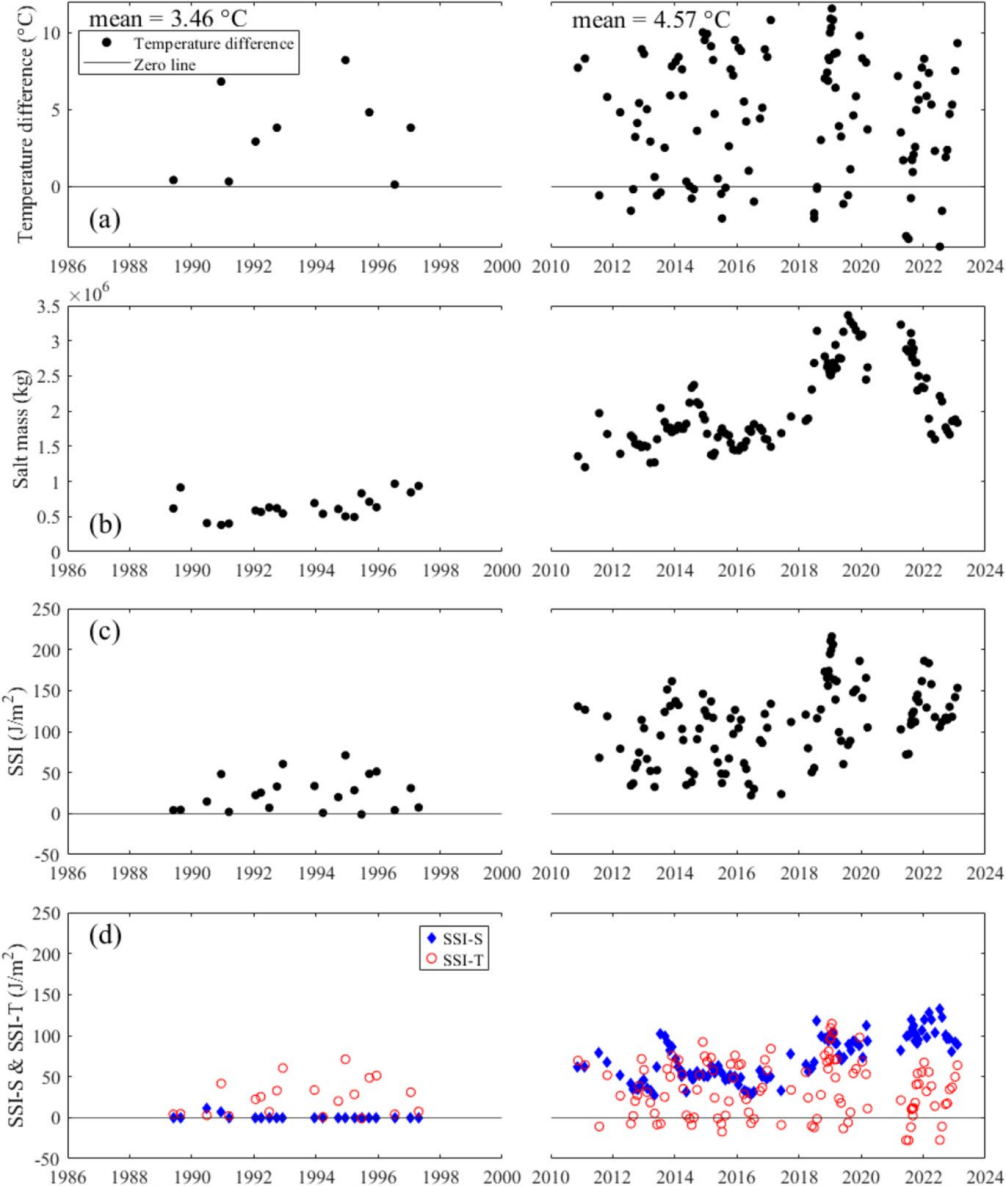
**a** Water temperature difference between 0.3 and 10 m depth. For data from 1986 to 1997, if measurements at 10 m were unavailable but deeper measurements were present, the deepest available value was used instead. **b** Total mass of salt. **c** Schmidt stability index for Lake Hugh Muntz during the study period. **d** Water temperature normalized Schmidt stability index (SSI-S) with a reference temperature of 20 °C, and the effect of water temperature on stability (SSI-T)

The Schmidt stability index (SSI) was calculated using Eq. 2 for periods 1 and 3 (see Table 1; period 2 is not included because data for depths > 6 m were not obtained). The total mass of salt in the lake increased, with a peak in 2019 of 3363 t, nearly nine times greater than the lowest mass of 380 t in 1990. SSI was < 5 $\text{J}/{\text{m}}^{2}$ during winter in the first seven years following construction and did not exceed 30 during summer. SSI increased markedly between the two calculated periods (Fig. 10). The average water temperature difference between the water surface (0.3 m) and bottom (10 m) increased by 1.05 °C from period 1 to period 3. This accounted for approximately 0.22 $\text{kg}/{\text{m}}^{3}$ of density difference between the two periods if salinity had remained constant. The temperature-normalized Schmidt Stability Index (SSI-S) indicates that the salinity gradient has been steadily increasing LHM’s stability since 2010. By the early 2020 s, the influence of salinity on SSI had nearly doubled compared to a decade earlier.

## Discussion

### Effects of saltwater intrusion

Saltwater intrusion will cause a freshwater lake’s salinity to elevate, creating a persistent density gradient, thus affecting the lake’s existing hydrodynamics (Sibert et al., 2015). Saltwater can move into a lake in different ways. In Sibert et al. (2015)’s case, the salt originated from high-salinity surface runoff from road salt application during winter, while in King and Tyler (1981) and Hodgson et al. (1997)’s study, groundwater seepage was the main source for Lake Fidler, Lake Morrison and Sulphide Pool of the lower Gordon River estuary.

LHM’s salinity increased after the expansion of the adjacent canal system in 1989. During the construction of the canal to the south of the lake, the previous stormwater overflow pipe was made bidirectional, allowing high-salinity canal water to flow into the lake during high tides. The salinity of the canal water ranges between 16.9 and 20 PSU (Macklin et al., 2017). The thermal stratification of the LHM almost continuous after the canal with high-salinity water was connected to the LHM. The stratifications were observed prior to 2010; however, it is difficult to ascertain whether the change was gradual or related to specific events due to the lack of the vertical profiles from 1997 to 2010. The intrusion of saline water has caused the salinity in bottom waters to build up, and the total amount of salt in the lake to be approximately nine-fold higher over the sampling period. As a result, vertical density gradients have reinforced summer thermal stratification, and the lake is now continuously density stratified.

LHM developed its meromixis because of regular saline water inundation. Based on this nature, we categorize the lake as Class A: Ectogenesis, Type I according to Walker and Likens’s (1975) meromictic lake nomenclature. It is worth noting that, Boehrer and Schultze (2008) argued that once a lake’s meromixis had formed, there could be various mechanisms that sustained the meromixis. Therefore, a meromictic lake’s classification may change from one type to another, but so far, we believe that intermittent saltwater intrusion from the exchange pipe is the main if not sole cause for LHM’s meromixis. However, exact whether or not or how much groundwater is in play remains unclear at this stage.

### Water quality changes

LHM has a mixolimnion that extends from the surface to a depth of approximately 6 m. The prolonged stratification has resulted in a permanently anoxic zone at depths > 6.0 m. This, in turn, influences biochemical processes in the lake, leading to a vertical pH gradient within the water column. In the mixolimnion, where phytoplankton flourishes, the pH tends to be above 7 due to ${CO}_{2}$ reduction because of photosynthesis, while in the monimolimnion, temperature is lower than the surface and ${CO}_{2}$ concentration is higher, resulting in a lower pH (Kalff, 2002).

The gradients of both DO and pH indicate the presence of a chemocline below the surface mixing layer, providing further evidence of the lake’s meromictic state (Boehrer & Schultze, 2008). Meromixis lakes have distinct geo-chemical processes compared with lakes that fully mix. Yang et al. (2020) stated that meromixis drove anoxia at monimolimnion, and the high salinity monimolimnion layer blocked the organic matter in sediment from contacting with the oxygen in the mixolimnion. Balistrieri et al. (1994) concluded that the concentration of sulphide and oxidation change at the chemocline causing concentrations of elements such as As, Co, Fe, Mn, Sb and etc. to vary across the oxic-anoxic interface.

High nutrient concentrations were observed in bottom waters of the lake below the chemocline, with concentrations of both total nitrogen and total phosphorus 10 times higher than those in surface waters. This is similar to other meromictic lakes such as Lake Starodworskie (Tandyrak et al., 2021) and Lake Burgsee (Fuchs et al., 2022).

Surface layer water quality data of LHM does not indicate a strong eutrophication state; however, the turbidity data also show a relatively high turbidity layer around 6.0 m, located at the bottom of the surface mixing layer. Coincidentally, chl-a measurements of the entire water column peak around this 6-m layer. We hypothesise that the turbidity maxima may indicate the presence of a particulate organic matter (POM) layer existing below the mixolimnion (a similar case can be observed in Lake Cadagno; see Danza et al. (2017); Saini et al. (2022); Tonolla et al. (2017)). This POM layer likely contains anoxygenic phototrophic sulphur bacteria, which are not uncommon in meromictic lakes (Zadereev et al., 2017). However, to confirm the existence of such bacteria in the water column, 16S rDNA and typical pigment identification are necessary (Muyzer & Stams, 2008).

LHM exhibited acidic water (pH < 4) during the first several years after its construction (Fig. 3 c). The likely cause of this initial acidic condition was the disturbance of acid sulphate soil during the lake’s construction. The Gold Coast area has a layer of pyrite that was laid down during the Holocene era, about 6000 years ago, when sea levels were higher. As sea levels receded, new sediments were deposited over this layer (Indraratna et al., 2002). This acid-sulphate soil layer is buried at elevations below 5 m AHD (Australian Height Datum) (Ahern, 2003). During the construction of the lake, the pyrite layer was likely disturbed and exposed to oxygen, leading to the formation of sulphuric acid from pyrite that subsequently led to acidic conditions in the first few years after lake filling. A sediment sampling campaign in LHM revealed that some sediment samples contained up to one-eighth of their dry weight as iron (Waltham et al., 2014), indicating that the lake was likely constructed in an area previously covered by a pyrite layer, which is composed of ferric sulphate.

### Surface water temperature trends

The model of surface temperature trend (Eq. 5) gave a rate of increase of 0.019 °C/year (Eq. 5). The increases in LHM’s surface water increase rate lies between the annual average increase in air temperature and ocean surface temperature from 1979 to 2012, which are 0.025 °C and 0.012 °C, respectively (IPCC, 2014). This value is consistent with the 50-year (1973–2022) trend of global surface temperature increase of 0.019 ± 0.001 °C/year suggested by Samset et al. (2023), but less than the rate of increase of surface water temperature for 235 lakes across the globe of 0.034 °C/year reported by O’Reilly et al. (2015). This suggests that the surface water temperature increase of LHM reflects a broader global warming signal, aligning with long-term global trends while exhibiting regional characteristics. Its warming rate is lower than the global average for lakes, which may indicate the presence of a buffering mechanism in the region. This could be attributed to the influence of the subtropical coastal climate, or potentially to the aforementioned canal modification that enhanced the lake’s connectivity with the ocean.

### LHM management and future research

The primary source of salinity in the lake has been identified as the exchange pipe connecting the lake to the adjacent canal. As water exchange between the lake and the canal becomes increasingly active, the vertical stratification stability of the lake is expected to further strengthen, making complete mixing more difficult to achieve. The currently observed retention of nutrients and hypoxic conditions in the bottom layer of the lake are projected to intensify over time. Climate change is expected to alter existing lake stratification patterns by increasing water column stability, potentially leading to the development of meromictic conditions in freshwater lakes (Woolway & Merchant, 2019; Woolway et al., 2021). A shift from monomictic to meromictic conditions can lead to prolonged hypoxia and nutrient accumulation in the bottom waters. If such a meromictic system is suddenly disrupted by extreme weather events, the resulting collapse of stratification may trigger an abrupt and potentially severe ecological disturbance—unlike the predictable, annual mixing seen in monomictic lakes. (Melack et al., 2017; Williams, 2002). These changes pose significant challenges for the protection of lake water quality and ecosystem health. Additionally, climate change, including global warming and sea-level rise, is expected to strengthen the stratification by intensifying the thermal gradient and increasing saline water input.

Therefore, we propose that management of lakes similar to LHM should emphasize the following key directions: (1) Managing the lake’s mass balance for both water and salt by regulating freshwater inflow and lake discharge. This includes increasing freshwater input to raise the lake’s water level, thereby reducing saline intrusion. Additionally, controlling saline water inflow is essential to limit salt accumulation. In the case of LHM, installing a flap valve on the connecting pipe can help regulate inflow, preventing excessive saline water from entering the lake. (2) Closely monitoring the vertical distribution of salinity, temperature, dissolved oxygen, and nutrients to enable early warning of chemocline shifts and eutrophication risks; (3) Introducing artificial stratification-disruption technologies when necessary, such as artificial aeration, to improve water column mixing, eliminate hypoxia, and alleviate nutrients retention in the bottom layer; and (4) Incorporating climate change as a critical contextual factor in lake management, accounting for the combined effect of sea level rise and thermal structure alterations in medium and long-term planning.

## Conclusion

Lake Hugh Muntz is a coastal artificial urban lake which has become meromictic in the past 14 years due to saltwater intrusion from adjacent saline water canals, likely associated with high tides. These inflows had neutralised the lake’s previously acidic state, which was likely started by the disturbance of a pyrite layer during the lake’s construction. The lake has since become productive, to an extent that surface waters are alkaline with surface pH around 8 to 10. The inorganic carbon cycle is also affected by phytoplankton activity.

The meromixis has led to typical characteristics of a meromictic lake, with distinct chemical properties between the mixolimnion and the monimolimnion, including higher nutrient content in the anoxic bottom water. A deep chlorophyll layer occurs between depths of approximately 4 and 6 M. Identification of the microbial community by sampling and DNA identification is necessary for confirming the existence of photosynthetic bacteria around the chemocline.

Being an urban lake in coastal regions, LHM faces challenges such as increasing anthropogenic activities and climate change impact. Warming temperature, higher sea levels, as well as increasing nutrient loading and lake productivity are all expected to affect the lake. Therefore, tools such as numerical modelling need to be considered to analyse and predict the future of LHM under climate change effects.

## Data Availability

The data supporting the findings of"Changes in Hydrodynamics and Water Quality of a Subtropical Meromictic Urban Lake"are available upon request via the following link. https://www.researchgate.net/publication/390704513.

## Declarations

All authors have read, understood, and have complied as applicable with the statement on “Ethical responsibilities of Authors” as found in the Instructions for Authors.
